# A Composite Substrate of Ag Nanoparticle-Decorated Inverse Opal Polydimethylsiloxane for Surface Raman Fluorescence Dual Enhancement

**DOI:** 10.3390/polym17141995

**Published:** 2025-07-21

**Authors:** Zilun Tang, Hongping Liang, Zhangyang Chen, Jianpeng Li, Jianyu Wu, Xianfeng Li, Dingshu Xiao

**Affiliations:** 1 School of Chemistry and Materials Engineering, Huizhou University, Huizhou 516007, China; tangzl@hzu.edu.cn (Z.T.); 18928934633@163.com (Z.C.); lijp@hzu.edu.cn (J.L.); lxfeng@hzu.edu.cn (X.L.); 2 Guangdong Provincial Key Laboratory of Electronic Functional Materials and Devices, Huizhou University, Huizhou 516007, China; 3 Science and Technology on Reliability Physics and Application of Electronic Component Laboratory, China Electronic Product Reliability and Environmental Testing Research Institute, Guangzhou 511370, China; wujianyu@ceprei.com

**Keywords:** surface-enhanced Raman scattering, surface-enhanced fluorescence, polymer-based composite substrate, Ag nanoparticle, inverse opal structure

## Abstract

It is difficult to simultaneously achieve surface-enhanced Raman scattering (SERS) and surface-enhanced fluorescence (SEF) for noble metals. Herein, a composite substrate is demonstrated based on the rational construction of Ag nanoparticles (Ag NPs) and inverse opal polydimethylsiloxane (PDMS) for surface Raman fluorescence dual enhancement. The well-designed Ag nanoparticle (Ag NP)-decorated inverse opal PDMS (AIOP) composite substrate is fabricated using the polystyrene (PS) photonic crystal method and the sensitization reduction technique. The inverse opal PDMS enhances the electromagnetic (EM) field by increasing the loading of Ag NPs and plasmonic coupling of Ag NPs, leading to SERS activity. The thin shell layer of polyvinyl pyrrolidone (PVP) in core–shell Ag NPs isolates the detected molecule from the Ag core to prevent the fluorescence resonance energy transfer and charge transfer to eliminate fluorescence quenching and enable SEF performance. Based on the blockage of the core–shell structure and the enhanced EM field originating from the inverse opal structure, the as-fabricated AIOP composite substrate shows dual enhancement in surface Raman fluorescence. The AIOP composite substrate in this work, which combines improved SERS activity and SEF performance, not only promotes the development of surface-enhanced spectroscopy but also shows promise for applications in flexible sensors.

## 1. Introduction

Surface-enhanced spectroscopy is a highly important technology in analysis; it is essential for environmental monitoring [1,2], biochemical detection [3,4,5], and food safety [6,7]. Surface-enhanced Raman scattering (SERS) and surface-enhanced fluorescence (SEF) are two types of surface-enhanced spectroscopy that offer the benefits of high sensitivity, anti-photobleaching, and anti-interference [8,9,10,11,12,13]. In practical applications, SERS and SEF technologies are more competitive and promising than other surface-enhanced spectroscopy methods.

To the best of our knowledge, the electrons on the surface of noble metals are able to generate surface plasmon resonance (SPR) with the incidence of external light, leading to an improved electromagnetic (EM) field on the surface of noble metals [14,15,16]. The improved EM field can greatly increase the Raman scattering and fluorescence intensity of the detected molecules [17,18,19,20]. Theoretically, noble metals are considered ideal candidates for SERS and SEF technologies. Because Ag has the best SPR, it is the most widely used noble metal at present. To further improve the EM field, the optimization of noble metals’ nanostructure and the composite of multiple noble metals are applied to improve plasmonic coupling [10,18,21,22,23]. In addition, noble metals have been incorporated into flexible substrates, such as PDMS [24,25,26], polyurethane (PU) [14,27], and poly (vinyl alcohol) (PVA) [28,29,30], to extend the application field in flexible sensors.

Generally, the detected molecules contact the surface of noble metals directly, which is beneficial for SERS but unfavorable for SEF. More specifically, the direct contact between detected molecules and a noble metal can promote their charge transfer, which can increase the Raman scattering intensity of the detected molecules [31,32]. However, fluorescence resonance energy transfer and charge transfer occur between detected molecules and noble metals because of their direct contact, leading to fluorescence quenching instead of enhanced fluorescence [33,34,35,36]. The noble metals that exhibit improved SERS activity tend to show limited SEF performance. To ensure improved SEF performance, an insulating layer was introduced to the surface of noble metals to avoid the occurrence of fluorescence resonance energy transfer and charge transfer [37,38,39]. However, the introduction of this layer inhibits the charge transfer between the detected molecules and the noble metal, reducing the SERS activity [40,41]. Thus, most studies only focus on SERS activity or SEF performance, and there are few reports regarding noble metals for surface Raman fluorescence dual enhancement. The development of strategies to endow noble metals with both improved SERS activity and SEF performance can promote the development of surface-enhanced spectroscopy.

**Scheme 1:**
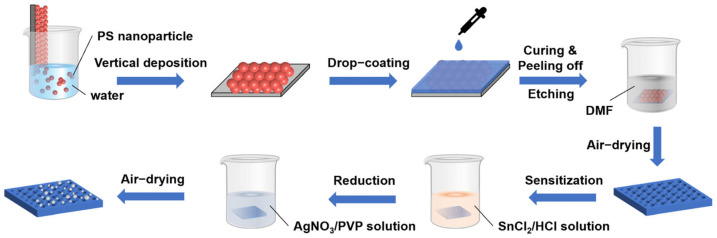
Schematic process of fabrication of AIOP composite substrate.

In this work, a sacrificial polystyrene (PS) photonic crystal method was employed to fabricate the inverse opal PDMS, and the surface was decorated with Ag nanoparticles (Ag NPs) using the sensitization reduction method to obtain a Ag nanoparticle (Ag NP)-decorated inverse opal PDMS (AIOP) composite substrate. As shown in Scheme 1, a glass wafer with air plasma treatment was vertically placed into a diluted PS nanoparticle suspension. As the water solvent evaporated, the PS nanoparticles were deposited onto the surface of the glass wafer to form highly ordered PS photonic crystals. PDMS was selected as the flexible polymer substrate and drop-coated onto the surface of PS photonic crystals. After curing and peeling off, the PS/PDMS composite film was immersed in a solution of N, N-dimethylformamide (DMF) to etch the PS nanoparticles. The sample was removed and air-dried to obtain an inverse opal film. The obtained film was immersed in SnCl_2_/HCl solution to anchor Sn^2+^ onto its surface. After that, the inverse opal film with the surface-anchoring Sn^2+^ was immersed in a AgNO_3_/PVP solution for the growth of Ag NPs. Finally, the sample was removed, cleaned with deionized water, and air-dried to obtain the AIOP composite substrate. Because of the increased loading of Ag NPs onto the inverse opal structure and their improved plasmonic coupling from Ag NPs, as well as the fluorescence quenching prevented by their core–shell structure, the AIOP composite substrate exhibited surface Raman fluorescence dual enhancement, offering ideas for the study of noble metal-based dual-enhanced spectroscopy.

## 2. Materials and Methods

### 2.1. Materials

Styrene, methylacrylic acid (MAA), potassium persulfate (KPS, 99.5%), polyvinyl pyrrolidone (PVP, 58000, K29-32), stannous chloride (SnCl_2_, 99%), Rhodamine 6G (R6G), malachite green (MG), and crystal violet (CV) were purchased from Shanghai Macklin Biochemical Technology Co., Ltd. (Shanghai, China). PDMS prepolymer with curing agent (DC 184) was obtained from Dow Corning (Midland, MI, USA). Sodium dodecyl sulfate (SDS, ≥90.0%) and silver nitrate (AgNO_3_, ≥99.8%) were purchased from Sinopharm Chemical Reagent Co., Ltd. (Shanghai, China). Hydrochloric acid (HCl) and N, N-dimethylformamide (DMF, 99.5%) were obtained from Guangzhou Chemical Reagent Factory (Guangzhou, China). Regioregular poly (3-hexylthiophene) (P3HT) was purchased from Luminescence Technology Corp (Hsinchu, China). All reagents and materials were used as received without further purification.

### 2.2. Synthesis of PS Nanoparticles

PS nanoparticles were synthesized according to our previous study [42]. Specifically, MAA (0.045 mL) and SDS (15 mg) were dissolved in deionized water (60 mL). When the above mixed solution was heated to 60 °C, styrene monomers were added and stirred for 30 min. The mixed solution was further heated to 80 °C. After that, 0.1 g/mL KPS aqueous solution was slowly added to the mixed solution and stirred for 10 h at 80 °C to obtain PS nanoparticles.

### 2.3. Preparation of PS/PDMS Composite Film

A total of 200 μL PS nanoparticle solution was added to 60 mL deionized water and stirred well to form dilute PS solution. The glass wafer was treated with air plasma for 10 min and then vertically placed into the dilute PS solution. As the water solvent evaporated, PS nanoparticles formed into a highly ordered nanostructure on the surface of the glass wafer. The mixture of PDMS prepolymer and curing agent in a ratio of 10:1 in mass was dropped onto the prepared highly ordered PS nanostructure. After curing at 60 °C in a vacuum oven for 12 h, the PS/PDMS composite film was obtained by peeling it off.

### 2.4. Fabrication of AIOP Composite Substrate

PS/PDMS composite film was immersed in DMF solution for 2 h. Based on the etching of DMF on PS, the inverse opal PDMS was obtained. Then, the inverse opal PDMS was removed from the DMF solution and dried naturally. In accordance with the sensitization reduction method in previous work [43], the resultant inverse opal PDMS was first immersed in SnCl_2_/HCl solution (0.02 mol/L) for 1 min and then immersed in Ag NO_3_/PVP solution (0.01 mol/L) for 1 min. Finally, the AIOP composite substrate was obtained by cleaning it with deionized water and air-drying.

### 2.5. Characterization

The morphologies of PS nanoparticles, the PS/PDMS composite film, the inverse opal PDMS, and the AIOP composite substrate were observed with a scanning electron microscope (SEM, SU8010, Hitachi, Tokyo, Japan). The morphologies of Ag NPs and their crystal structures were measured using a transmission electron microscope (TEM, JEM-F200, JEOL, Tokyo, Japan). The chemical structure of PS nanoparticles was characterized with a Fourier transform infrared (FTIR) spectrometer (IS50R, Thermo Fisher, Waltham, MA, USA). The cavity depth of the inverse opal PDMS was determined with atomic force microscopy (AFM, Dimension FastScan, Bruker, Billerica, MA, USA). The surface analysis of the AIOP composite substrate was investigated using X-ray diffraction (XRD, miniflex600, Rigaku, Tokyo, Japan) with Cu Kα radiation at a Bragg angle ranging from 10 to 90° and X-ray photoelectron spectroscopy (XPS, Escalab 250Xi, Thermo Fisher, Waltham, MA, USA). The UV-Vis spectra of the AIOP composite substrate and regioregular P3HT were obtained using an ultraviolet spectrophotometer (UV-2600i, Shimadzu, Kyoto, Japan). The reflection spectra of PS nanoparticles after self-assembly and the PS/PDMS composite were obtained with a fiber spectrometer (PG2000-Pro-EX, Shanghai Fuxiang Optics Co., Ltd., Shanghai, China). 

### 2.6. SERS and SEF Measurement

R6G was dissolved in deionized water to obtain aqueous solutions of different concentrations, from 1 × 10^−4^ M to 1 × 10^−7^ M, while MG and CV were dissolved in the deionized water to form an aqueous solution with a concentration of 1 × 10^−4^ M. The Raman spectra of R6G, MG, and CV on pure PDMS, Ag NP-decorated PDMS, and the AIOP composite substrate were collected using a micro confocal Raman spectrometer (in Via, RENISHAW, Wotton-under-Edge, UK) with a 633 nm excited laser and a 50× objective lens. The Raman measurements were performed with a 10 s acquisition time for one accumulation. A 633 nm excited laser was focused on a random area of 25 × 25 μm^2^ using a 50× objective lens, and the peak of R6G at 1360 cm^−1^ was selected as the characteristic Raman signal for Raman mapping. The acquisition time was 10 s for one accumulation, and Raman mapping with a solution of 5 × 5 pixels was obtained. The fluorescence emission spectra of regioregular P3HT on the samples were measured with a fluorescence spectrometer (FLS1000, Edinburgh Instruments, Livingston, UK) ranging from 680 to 800 nm. A Xe lamp was used as the excited source, and its wavelength was set to 448 nm.

### 2.7. EM Field Simulation

EM field distributions among core–shell Ag NPs were calculated using COSMOL Multiphysics. Simulation models were constructed of the decoration of Ag NPs with a diameter ranging from 10 to 50 nm on the inverse opal PDMS and flat PDMS, which were arranged in the x–z plane. The wavelengths of incident plane waves were set at 633 and 448 nm, and the incident plane waves were polarized along the *x*-axis and incident along the *z*-axis.

## 3. Results and Discussion

### 3.1. Characterization of PS Nanoparticles

**Figure 1:**
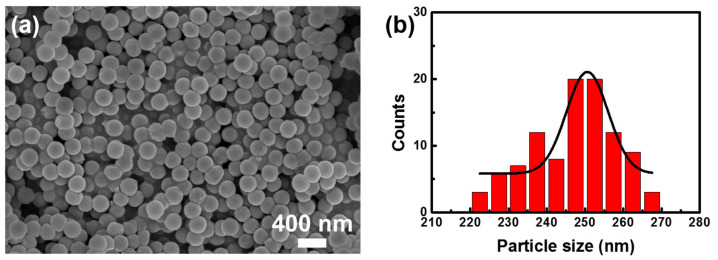
(**a**) SEM image and (**b**) size distribution histogram of PS nanoparticles.

The morphology of as-synthesized PS nanoparticles is shown in Figure 1a. The PS nanoparticles had a spherical shape with a uniform particle size. Without any post-treatment, the as-synthesized PS nanoparticles were in a disordered arrangement. According to the size distribution histogram in Figure 1b, the diameter of the PS nanoparticles was 251 nm.

To reveal the chemical structure of the PS nanoparticles, we used FTIR spectrometry (Figure S1). The absorption peaks at 1600, 1492, and 1450 cm^−1^ were attributed to the bending vibrations of the benzene ring skeleton. The absorption peaks at 3442, 1381, 912, and 1697 cm^−1^ were attributed to the stretching vibrations of -OH and -C=O in carboxyl groups [44]. These results confirmed that PS nanoparticles were successfully synthesized.

### 3.2. Self-Assembly and Transfer of PS Nanoparticles on PDMS

**Figure 2:**
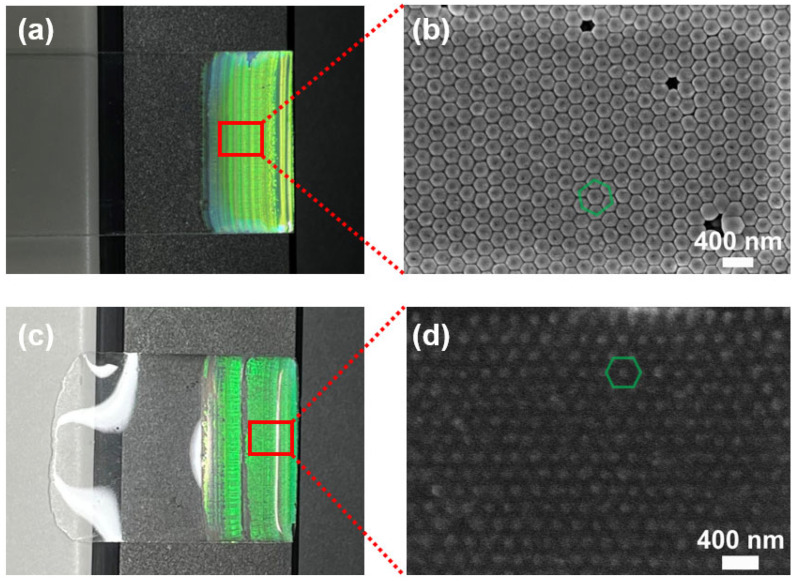
(**a**) Photograph and (**b**) SEM image of PS nanoparticles after self-assembly; (**c**) photograph and (**d**) SEM image of PS/PDMS composite film.

The PS photonic crystals on the glass wafer were formed via the self-assembly of vertical deposition, which shows an obvious structural color of green (Figure 2a). The reflection peak of the PS photonic crystals on the glass wafer was located at 526 nm (Figure S2a), which was within the wavelength range of green light [45]. Figure 2b shows an SEM image of the PS nanoparticles after self-assembly. They were arranged in an ordered hexagonal structure. As a result, Bragg diffraction was achieved, leading to the green structural color.

In this study, PDMS was chosen as the flexible substrate for PS photonic crystals. After transferring the PS photonic crystals onto PDMS, the PS/PDMS composite showed a green structural color (Figure 2c), and its reflection peak was in the wavelength range of green light, as before (Figure S2b). Figure 2d presents an SEM image of the PS/PDMS composite film. Each PS nanoparticle was partly embedded in the PDMS, while the remaining portion was exposed to the outside. The PS nanoparticles remained in a hexagonal arrangement, which indicated that the ordered hexagonal structure of the PS photonic crystals was not affected by the introduction of PDMS. All of these results confirmed that the PS nanoparticles with an ordered hexagonal arrangement were successfully transferred onto the PDMS substrate. Although PS photonic crystals had been transferred onto the PDMS substrate, the glass wafer after the transfer of PS photonic crystals still exhibited a green structural color (Figure S3), indicating that the transfer was incomplete.

### 3.3. Characterization and Surface Analysis of AIOP Composite Substrate

**Figure 3:**
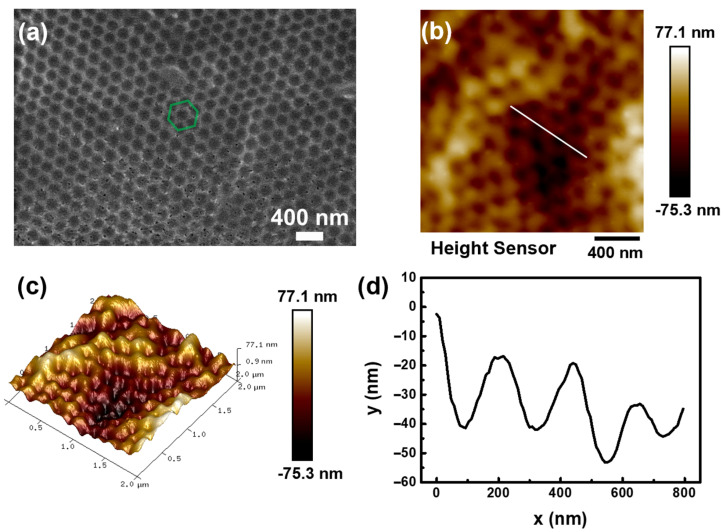
(**a**) SEM, (**b**) 2D, and (**c**) 3D AFM height images of the inverse opal PDMS; (**d**) roughness variation along the white line indicated in (**b**).

For PS/PDMS composite film, DMF is a good solvent for PS, but it does not provide a solvent for PDMS. Therefore, PS can be selectively etched with DMF to obtain the inverse opal PDMS, which is shown in Figure 3a. A monolayer of cavities was observed on the surface of PDMS. The cavities were still arranged in an ordered hexagonal structure, which illustrated that the arrangement of the PS nanoparticles was not destroyed by DMF during etching. The refractive index of air is 1.0, while that of PDMS is 1.4. There is a small refractive index difference (Δ*n* = 0.4) between air and PDMS, which has an adverse effect on Bragg diffraction, resulting in weakened reflection. In addition, the obtained inverse opal structure consisted of cavities instead of complete spherical structures, which also resulted in weakened reflection. Thus, the inverse opal PDMS had no structural color, which is shown in the inset of Figure S4.

To measure the diameter and depth of the cavities, a roughness test was carried out. Figure 3b,c present 2D and 3D AFM height images of the inverse opal PDMS. The spherical dispersed phase consisted of cavities resulting from the etching of DMF on PS nanoparticles, while the continuous phase was a PDMS matrix. Figure 3d shows the roughness variation along the white line indicated in Figure 3b. The average diameter of the cavities was 200 nm—smaller than the 251 nm diameter of the PS nanoparticles—with an average depth of 40 nm. These results confirmed that the inverse opal PDMS was successfully fabricated.

**Figure 4:**
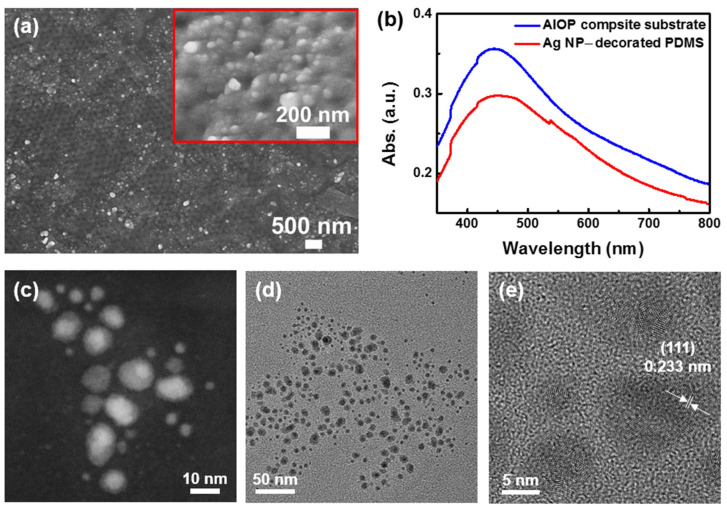
(**a**) SEM image of AIOP composite substrate; (**b**) absorption spectra of AIOP composite substrate and Ag NP-decorated PDMS; (**c**) STEM, (**d**) TEM, and (**e**) HRTEM images of Ag NPs from AIOP composite substrate.

As shown in Figure S5, only parts of the flat PDMS surface were decorated with Ag NPs. Because of its hydrophobicity [46,47], the flat PDMS could not be wetted by the Ag growth solution, leading to only parts of spots being decorated. Figure 4a shows the morphologies of the AIOP composite substrate. More spots of the inverse opal PDMS were decorated with Ag NPs because the inverse opal PDMS provided a higher surface area for the wetting of the Ag growth solution. Thus, the inverse opal structure was able to improve the loading of Ag NPs. Despite the higher surface area in contact with the Ag growth solution, certain spots remained uncovered by Ag NPs on the inverse opal PDMS surface. As shown in the inset of Figure 4a, Ag NPs with diameters ranging from 10 to 50 nm were distributed on the outer surface of cavities, whereas no Ag NPs were found on the inner surface of cavities. The Ag growth solution could not penetrate into the inner surface of the cavities due to its hydrophobicity in relation to PDMS. Instead, the Ag growth solution could only enrich near the rim of the cavities, leading to the aggregation of Ag NPs on their outer surface. Figure 4b shows the absorption spectrum of the AIOP composite substrate and Ag NP-decorated PDMS. The absorption peak at 450 nm was detected and attributed to the plasmon resonance of Ag NPs [48]. In comparison, the intensity of the plasmonic peak for the AIOP composite substrate was stronger than the Ag NP-decorated PDMS. Figure 4c, d present STEM and TEM images of Ag NPs from the AIOP composite substrate, which indicate that the Ag NPs possess a core–shell structure. During the fabrication of the AIOP composite substrate, PVP was introduced to the oxidation reduction of Ag NPs. On the one hand, PVP played an important role in protecting Ag NPs. On the other hand, PVP was able to facilitate the anchoring of Sn^2+^ onto the surface of Ag NPs, which was beneficial for the growth of Ag NPs. Therefore, the Ag NPs were encapsulated by the thin layer of PVP. As shown in Figure 4e, the d-spacing of the lattice fringe of the Ag NPs was 0.233 nm, which was attributed to the (111) plane of Ag NPs [49].

**Figure 5:**
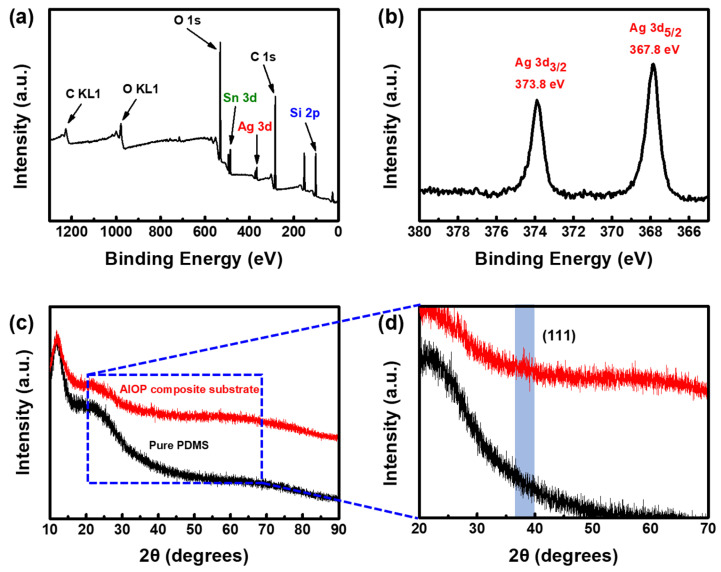
(**a**) XPS survey spectrum of AIOP composite substrate; (**b**) high-resolution XPS spectrum of Ag 3d binding energy; (**c**) XRD patterns of pure PDMS and AIOP composite substrate; (**d**) enlargement of the XRD patterns at specified positions for pure PDMS and AIOP composite substrate.

The surface elements of the AIOP composite substrate were measured with XPS. Figure 5a shows the XPS survey spectrum of the AIOP composite substrate. The C, O, Si, Ag, and Sn elements were detected. The C, O, and Si elements originated from the PDMS, while the Ag element originated from the Ag NPs. Because Ag NPs were prepared from the oxidation of Sn^2+^, the appearance of the Sn element on the AIOP composite substrate was inevitable. Figure 5b shows the high-resolution XPS spectrum of the Ag 3d binding energy. Two peaks were detected at the positions of 373.8 and 367.8 eV, which corresponded to the peaks of Ag 3d3/2 and Ag 3d5/2, respectively [50]. Figure 5c,d present the XRD patterns of pure PDMS and AIOP composite substrate. In comparison, there was a weak peak at 38.15° for the diffraction pattern of the AIOP composite substrate, which corresponded to the (111) fraction direction of the Ag NPs [51], agreeing with the test results from HRTEM images of Ag NPs from AIOP composite substrate (Figure 4e). Because of the small size and core–shell structure of Ag NPs, only the diffraction peak of the (111) plane was detected in the XRD pattern of the AIOP composite substrate. The above SEM, STEM, TEM, UV-Vis, XPS, and XRD results confirmed the successful fabrication of AIOP composite substrate.

### 3.4. SERS Activity of AIOP Composite Substrate

**Figure 6:**
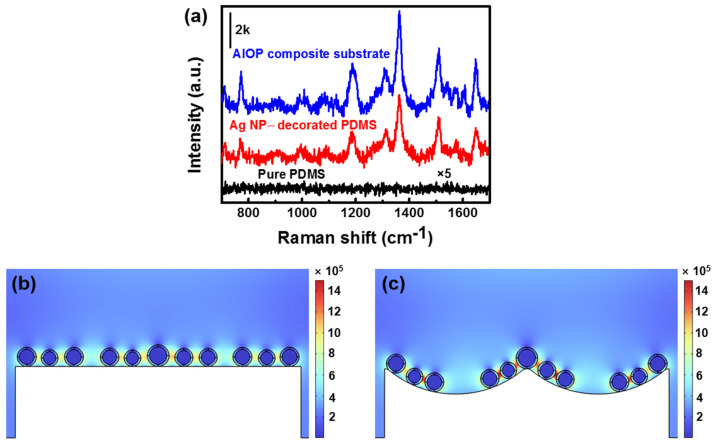
(**a**) Raman spectra of R6G (1 × 10^−4^ M) on pure PDMS, Ag NP-decorated PDMS, and AIOP composite substrate; EM field distributions of cross-section of (**b**) Ag NP-decorated PDMS and (**c**) AIOP composite substrate under excitation of 633 nm.

To evaluate the SERS activity of AIOP composite substrate, Raman measurements of R6G probe molecule (1 × 10^−4^ M) were conducted on pure PDMS, Ag NP-decorated PDMS, and AIOP composite substrate, and a 633 nm excited laser was selected to ensure the Raman signals of R6G and reduce the interference of its fluorescence in Raman scattering (Figure 6a). Notably, the Raman characteristic peaks of R6G were clearly observed on the substrates of Ag NP-decorated PDMS and AIOP composite substrate, whereas the Raman characteristic peaks of R6G were not detected on pure PDMS substrate, even when magnified five times. The Raman peaks at 770 and 1187 cm^−1^ were attributed to C−H out-plane bending and C−H/N−H xanthene ring bending, while the Raman peak at 1306 cm^−1^ was due to C=C stretching. The Raman peaks at 1360 and 1502 cm^−1^ were attributed to C−C xanthene ring stretching, while the Raman peak at 1650 cm^−1^ was a result of C−C in-plane stretching (Table S1) [52,53]. In comparison, the AIOP composite substrate exhibited greater SERS activity than the Ag NP-decorated PDMS.

As shown in Figure 4c,d, the Ag NP-decorated inverse opal PDMS exhibited a core–shell structure, where Ag served as the core and PVP served as the shell. The PVP shell increased the distance of Ag NPs, which resulted in decreased plasmonic coupling between adjacent Ag NPs. As shown in Figure 6b, plasmonic coupling still occurred between adjacent Ag NPs on the flat PDMS despite the presence of the PVP shell. Based on the inverse opal structure, together with the hydrophobicity of PDMS, Ag NPs aggregated near the outer surface of cavities. The spacing between the aggregated Ag NPs was reduced for the AIOP composite substrate, which improved the plasmonic coupling to enhance the overlap of the EM field between adjacent Ag NPs. As shown in Figure 6c, the AIOP composite substrate exhibited stronger plasmonic coupling compared with the decoration of Ag NPs on the flat PDMS. Because of the higher loading of Ag NPs and the stronger interparticle plasmonic coupling, the intensity of the plasmonic peak for the AIOP composite substrate was stronger than that of the Ag NP-decorated PDMS (Figure 4b), and Ag NPs on inverse opal PDMS exhibited greater SERS performance than those on flat PDMS (Figure 6a).

Typically, SERS enhancement mechanisms can be divided into the EM mechanism and the chemical mechanism (CM), where the EM mechanism originates from SPR in noble metals, while the CM mechanism relates to the charge transfer between the Raman probe molecule and the substrate surface [54]. The PVP shell blocked the charge transfer between Raman probe molecules and Ag NPs, which reduced the SERS activity via the CM mechanism. In contrast, the inverse opal structure from the AIOP composite substrate improved SERS activity via the EM mechanism [55]. It is well-established that the EM mechanism plays a predominant role in SERS activity compared with the CM mechanism. The inverse opal structure in the AIOP substrate improved SERS activity via increased loading of Ag NPs and enhanced plasmonic coupling between Ag NPs, compensating for SERS activity loss from the core–shell structure of Ag NPs. Therefore, the AIOP composite substrate still exhibited the improved SERS activity. In addition, the PVP shell was anchored onto the surface of Ag via strong chemical coordinate bonds, which prevented the whole PVP segment from detaching from the Ag NPs and dissolving [56,57]. Therefore, core–shell Ag NPs could stably exist in water, and the structural stability of the PVP shell layer remained unaffected during SERS measurements. We chose MG and CV as other analytes to investigate the SERS performance of pure PDMS, Ag NP-decorated PDMS, and the AIOP composite substrate (Figure S7). The Raman characteristic peaks of MG (916, 1171, 1294, 1365, 1589, 1641 cm^−1^) and CV (915, 1171, 1371, 1531, 1629 cm^−1^) were observed on the substrates of Ag NP-decorated PDMS and the AIOP composite substrate (Tables S2 and S3). Consistent with the results above, the AIOP composite substrate exhibited greater SERS activity.

To investigate the SERS sensitivity of the AIOP composite substrate, Raman measurements of R6G with serially diluted concentrations were carried out. As shown in Figure S3a, the Raman peaks at 770 and 1360 cm^−1^ were still observed even if the concentration of R6G decreased to 1 × 10^−7^ M. Thus, it can be concluded that the SERS sensitivity of the prepared AIOP composite substrate for R6G is 1 × 10^−7^ M. The Raman peak at 1360 cm^−1^ and the region of 25 × 25 μm^2^ from the AIOP composite substrate were selected for Raman mapping. Figure S3b shows the Raman mapping at a resolution of 5×5 pixels of R6G (1 × 10^−4^ M) on the AIOP composite substrate. Few parts of spots exhibited low Raman intensity with a black color, which was due to the uneven distribution of Ag NPs on the AIOP composite substrate (Figure 4a). Overall, most of the spots exhibited similar blue colors, which demonstrated that their SERS activities were close to each other.

### 3.5. SEF Performance of AIOP Composite Substrate

**Figure 7:**
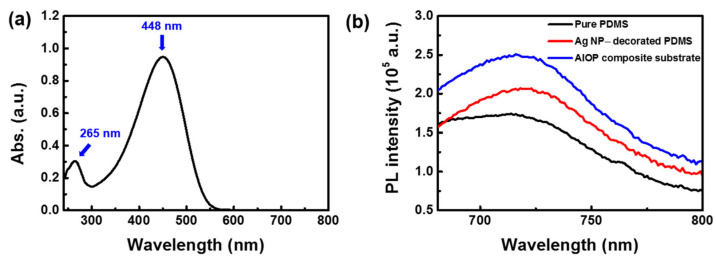
(**a**) Absorption spectrum of regioregular P3HT in trichloromethane; (**b**) fluorescence spectra of regioregular P3HT ranging from 680 to 800 nm on pure PDMS, Ag NP-decorated PDMS, and AIOP composite substrate.

In this study, regioregular P3HT was chosen as a fluorescence probe molecule to investigate SEF performance. Figure 7a shows the absorption spectrum of regioregular P3HT. It can be seen that the peaks at 265 and 448 nm appeared in this spectrum. The peak at 448 nm was stronger and very close to the plasmonic resonance peak of Ag NPs (Figure 4b), which was favorable for the improvement of the regioregular P3HT fluorescence excited rate.

Regioregular P3HT was dissolved in trichloromethane to form a mixed solution (1 mg/mL) and then spin-coated on the AIOP composite substrate. The excitation wavelength at 448 nm was chosen for the fluorescence test of regioregular P3HT. Figure S4 presents the fluorescence spectra of the AIOP composite substrate. There were no peaks for this substrate, which indicated that fluorescence did not occur in it with a wavelength range from 680 to 800 nm. Figure 7b shows the fluorescence spectrum of regioregular P3HT on pure PDMS, Ag NP-decorated PDMS, and the AIOP composite substrate. The fluorescence emission peaks at 725 nm of regioregular P3HT were detected in all substrates, corresponding to the (0–1) transition of regioregular P3HT [58]. The fluorescence emission peak of regioregular P3HT was strongest on the AIOP composite substrate, followed by Ag NP-decorated PDMS, and weakest on pure PDMS.

The STEM and TEM images in Figure 4c,d indicate that the Ag NPs on the AIOP composite substrate had a core–shell structure, with Ag as the core and PVP as the shell. Because of the blocking of a thin layer of PVP, the fluorescence probe molecule P3HT was not directly in contact with Ag. There was no fluorescence resonance energy transfer or charge transfer between P3HT and Ag, avoiding the occurrence of fluorescence quenching. Under the excitation of 448 nm, strong plasmonic coupling still occurred between adjacent Ag NPs on the AIOP composite substrate. This plasmonic coupling was stronger than that of Ag NP-decorated PDMS, which is shown in Figure S5a,b. In addition, the inverse opal structure was able to improve the loading of Ag NPs. The blocking of PVP, the stronger plasmonic coupling, and the improved loading of Ag NPs improved the SEF performance of the AIOP composite substrate.

Overall, the Raman fluorescence dual enhancement of probe molecules of the AIOP composite substrate in this study can be achieved. In view of the combination of SERS activity and SEF performance, this work provides functional diversity for surface-enhanced spectroscopy. In addition, the AIOP composite substrate is fabricated based on a PDMS flexible polymer substrate. It is expected that this polymer-based material can be used in the practical application of flexible sensors.

## 4. Conclusions

In summary, we developed a polymer-based composite substrate of Ag NP-decorated inverse opal PDMS for surface Raman fluorescence dual enhancement. The AIOP composite substrate was fabricated using the sacrificial PS photonic crystals method and the sensitization reduction method. The inverse opal structure in the AIOP composite substrate improved the loading of Ag NPs and increased the plasmonic coupling of Ag NPs to compensate for the weakness in SERS activity originating from the core–shell structure of the Ag NPs, which improved the SERS activity of the AIOP composite substrate. Meanwhile, the core–shell structure of Ag NPs avoided the occurrence of fluorescence resonance energy transfer and charge transfer between detected molecules and Ag, which prevented fluorescence quenching, leading to SEF performance. The obtained AIOP composite substrate exhibited SERS activity and SEF performance simultaneously. This study is significant for the development of surface-enhanced spectroscopy and has promising application prospects in the field of flexible sensors.

## Data Availability

The original contributions presented in this study are included in the article. Further inquiries can be directed to the corresponding authors.

## Supplementary Materials

The following supporting information can be downloaded at https://www.mdpi.com/article/10.3390/polym17141995/s1: Figure S1: FTIR spectrum of PS nanoparticles; Figure S2: Reflection spectra of (a) PS nanoparticles after self-assembly and (b) PS/PDMS composite film; Figure S3: Photograph of the glass wafer after the transfer of PS photonic crystals; Figure S4: Photograph of the PS/PDMS composite film (a) before and (b) after the etching of DMF; Figure S5: SEM image of Ag NP-decorated PDMS; Figure S6: (a) Raman spectra of R6G with a concentration from 1 × 10^−4^ M to 1 × 10^−7^ M on the AIOP composite substrate; (b) Raman mapping at a resolution of 5 × 5 pixels of R6G (1 × 10^−4^ M) on the AIOP composite substrate; Figure S7: Raman spectra of (a) MG (1 × 10^−4^ M) and (b) CV (1 × 10^−4^ M) on pure PDMS, Ag NP-decorated PDMS, and AIOP composite substrate; Figure S8: Fluorescence spectra of AIOP composite substrate ranging from 680 to 800 nm; Figure S9: EM field distributions of cross-section of (a) Ag NP-decorated PDMS and (b) AIOP composite substrate under excitation of 448 nm; Table S1: Vibrational models of the Raman peaks for R6G molecules; Table S2: Vibrational models of the Raman peaks for MG molecules; Table S3: Vibrational models of the Raman peaks for CV molecules. References [52,53,59,60,61] are cited in the Supplementary Materials.

## References

[1] Li D., Wu X., Chen Z., Liu T., Mu X. (2025). Surface-enhanced spectroscopy technology based on metamaterials. Microsyst. Nanoeng..

[2] Srivastava S., Wang W., Zhou W., Jin M., Vikesland P.J. (2024). Machine learning-assisted surface-enhanced Raman spectroscopy detection for environmental applications: A review. Environ. Sci. Technol..

[3] Lu Y., Lin L., Ye J. (2022). Human metabolite detection by surface-enhanced Raman spectroscopy. Mater. Today Bio.

[4] Li Z., Chen M., Liang Q.-B., Pan X.-H., Wang J.-D., Weng Y.-H., Cao S.-H., Li Y.-Q. (2025). Covalent organic framework-based surface plasmon-enhanced fluorescence sensing for real-time monitoring of cell apoptosis. Biosens. Bioelectron..

[5] Berger L.M., Duportal M., Menezes L.d.S., Cortés E., Maier S.A., Tittl A., Krischer K. (2023). Improved In Situ Characterization of Electrochemical Interfaces Using Metasurface-Driven Surface-Enhanced IR Absorption Spectroscopy. Adv. Funct. Mater..

[6] Wu T., Mao Y., Wang T., Ma L., Cao J., Xie B., Weia J., Lia P. (2025). Evolving trends in nanozyme-based SERS systems for food contaminant monitoring: A review. Food Chem..

[7] Logan N., Cao C., Freitag S., Haughey S.A., Krska R., Elliott C.T. (2024). Advancing Mycotoxin Detection in Food and Feed: Novel Insights from Surface-Enhanced Raman Spectroscopy (SERS). Adv. Mater..

[8] Xu C., Chen S., Sun W., Xu R., Yin X., Guo D., Yang Y. (2025). Plasmon-enhanced fluorescence sensor based on Au nanocages for sensitive detection of norepinephrine. Anal. Chim. Acta.

[9] Ge Y., Wang H., Li Q., Li Q., Yang Y., Zhu R., Yang J., Liu X., Zhang Q., Wang Y. (2025). Rapid and Scalable Preparation of Highly Uniform, Atomically Thin MSe2 (M = Ti, Nb, Ta) Nanosheets as Ultra-Sensitive SERS Substrates for Lateral Flow Immunoassay. Adv. Funct. Mater..

[10] Huang X., Tian H., Huang L., Chen Q., Yang Y., Zeng R., Xu J., Chen S., Zhou X., Liu G. (2023). Well-ordered Au nanoarray for sensitive and reproducible detection of hepatocellular carcinoma-associated miRNA via CHA-assisted SERS/fluorescence dual-mode sensing. Anal. Chem..

[11] Hang Y., Boryczka J., Wu N. (2022). Visible-light and near-infrared fluorescence and surface-enhanced Raman scattering point-of-care sensing and bio-imaging: A review. Chem. Soc. Rev..

[12] Apostolaki M.-A., Sakellis E., Gardelis S., Likodimos V. (2025). Interplay of plasmonic and charge transfer effects for ultrasensitive Ag–WO 3/TiO 2 photonic crystal SERS sensors. Mater. Adv..

[13] Wang J., Pinkse P.W., Segerink L.I., Eijkel J.C. (2021). Bottom-up assembled photonic crystals for structure-enabled label-free sensing. ACS Nano.

[14] Tang Z., Wu J., Yu X., Hong R., Zu X., Lin X., Luo H., Lin W., Yi G. (2021). Fabrication of Au nanoparticle arrays on flexible substrate for tunable localized surface plasmon resonance. ACS Appl. Mater. Interfaces.

[15] Deka T., Nair R.G. (2024). Recent advancements in surface plasmon resonance and Schottky junction assisted photocatalytic water splitting of noble metal decorated Titania: A review. Int. J. Hydrogen Energy.

[16] Cai Y.Y., Choi Y.C., Kagan C.R. (2023). Chemical and Physical Properties of Photonic Noble-Metal Nanomaterials. Adv. Mater..

[17] Choi J.-H., Choi J.-W. (2020). Metal-enhanced fluorescence by bifunctional Au nanoparticles for highly sensitive and simple detection of proteolytic enzyme. Nano Lett..

[18] Wang D., Xu X., Qiu T., Ren N., Liu H., Fang G., Liu Z. (2025). Laser Construction TiOx-Ag heterostructure interface for high sensitive coupling resonance enhanced fluorescence in bacterial nucleic acid amplification sensor chip. Sens. Actuators B Chem..

[19] Lin L.L., Alvarez-Puebla R., Liz-Marzán L.M., Trau M., Wang J., Fabris L., Wang X., Liu G., Xu S., Han X.X. (2025). Surface-enhanced Raman spectroscopy for biomedical applications: Recent advances and future challenges. ACS Appl. Mater. Interfaces.

[20] Zhao J., Chen Y., Tang Y., Li B., Wang Q., Wang J., Gao X., Zhang Y., Wang J., Lei Z. (2025). Tandem Dual-Ratiometric SERS Probe Enables Raman Imaging of Neurological pH Fluctuations in Epilepsy. Angew. Chem. Int. Ed..

[21] Meng T., Shi M., Guo Y., Wang H., Fu N., Liu Z., Huang B., Lei C., Su X., Peng B. (2022). Multifunctional Ag-coated CuO microbowl arrays for highly efficient, ultrasensitive, and recyclable surface-enhanced Raman scattering. Sens. Actuators B Chem..

[22] Hu X., Mu Z., Li Y., Bai L., Qing M. (2025). Metal–organic frameworks-scaffold gold nanoclusters enabled aggregation-induced enhanced fluorescent sensor array for high-throughput detection of heavy metal ions. Microchem. J..

[23] Minamikawa T., Sakaguchi R., Harada Y., Tanioka H., Inoue S., Hase H., Mori Y., Takamatsu T., Yamasaki Y., Morimoto Y. (2024). Long-range enhancement for fluorescence and Raman spectroscopy using Ag nanoislands protected with column-structured silica overlayer. Light Sci. Appl..

[24] Luo J., Wang Z., Li Y., Wang C., Sun J., Ye W., Wang X., Shao B. (2021). Durable and flexible Ag-nanowire-embedded PDMS films for the recyclable swabbing detection of malachite green residue in fruits and fingerprints. Sens. Actuators B Chem..

[25] Li X., Liu H., Gu C., Zhang J., Jiang T. (2022). PDMS/TiO2/Ag hybrid substrate with intrinsic signal and clean surface for recyclable and quantitative SERS sensing. Sens. Actuators B Chem..

[26] Choudhary R., Srivastava S.K. (2025). Templating assisted fabrication of flexible, highly stable and uniform plasmonic platform for ultrahigh enhancement of Raman and fluorescence signals: Model sensing of rhodamine-6G. Spectrochim. Acta Part A Mol. Biomol. Spectrosc..

[27] Yi K., Fan Z., Ran Q., Jia K., Liu X., Wang L. (2023). Scalable fabrication of silver covered polyurethane nanofibers as flexible SERS nanosensors for aflatoxin detection. Talanta.

[28] Shao T., Xu J., Zhong H., Hu Y., Chen J. (2024). A stable and flexible Au@ Ag NPs/PVA SERS platform for thiram residue detection on rough surface. Talanta.

[29] Chang R., Wang T., Liu Q., Tang J., Wu D. (2022). Ag nanoparticles@ agar gel as a 3D flexible and stable SERS substrate with ultrahigh sensitivity. Langmuir.

[30] Zhao X., Zhang L., Lv X., Liu J., Liu X., Zhang Y., Zhang D., Li S., Wang Q. (2022). Large-area fluorescence enhancement of R6G based on a uniform PVA-Au plasmonic substrate. Opt. Express.

[31] Ma H., Pan S.-Q., Wang W.-L., Yue X., Xi X.-H., Yan S., Wu D.-Y., Wang X., Liu G., Ren B. (2024). Surface-enhanced Raman spectroscopy: Current understanding, challenges, and opportunities. ACS Nano.

[32] Tang X., Hao Q., Hou X., Lan L., Li M., Yao L., Zhao X., Ni Z., Fan X., Qiu T. (2024). Exploring and engineering 2D transition metal dichalcogenides toward ultimate SERS performance. Adv. Mater..

[33] Ma Y., Song M., Li L., Lao X., Liu Y., Wong M.-c., Yang M., Chen H., Hao J. (2024). Attomolar-level detection of respiratory virus long-chain oligonucleotides based on FRET biosensor with upconversion nanoparticles and Au–Au dimer. Biosens. Bioelectron..

[34] He S., Xiang H., Zhao G., Zhang M., Lin J., Yang L., Liu H. (2023). A FRET assembly of fluorescent gold nanoclusters for ratiometric monitoring peroxynitrite in living cells and zebrafish. Sens. Actuators B Chem..

[35] Yang N., Kang Y., Cong Y., Wang X., Yao C., Wang S., Li L. (2023). Controllable gold nanocluster–emulsion interface for direct cell penetration and photothermal killing. Adv. Mater..

[36] Lai X., Zhang G., Deng S., Zhang G., Xiao X., He W., Su L., Liu C., Lai W. (2023). Triple strategy-enhanced immunochromatographic assay based on APCB and AIEFM for the ultrasensitive detection of AFM1. J. Hazard. Mater..

[37] Huang K., Le N., Wang J.S., Huang L., Zeng L., Xu W.C., Li Z., Li Y., Han G. (2022). Designing next generation of persistent luminescence: Recent advances in uniform persistent luminescence nanoparticles. Adv. Mater..

[38] Cai Y., Lu M., Qin X., Jin D., Zhou J. (2025). Understanding shell coating effects to overcome quenching in single anisotropic upconversion nanoparticles. Nat. Commun..

[39] Qi B., Dai W., Lou B., Song B., Miao Z., Wei Y., Ma C., Wang J. (2025). Amplifying Persistent Luminescence in Heavily Doped Nanopearls for Bioimaging and Solar-to-Chemical Synthesis. ACS Nano.

[40] Zhu Y.-Z., Zhou R.-Y., Hu S., Li J.-F., Tian Z.-Q. (2024). Shell-Isolated Nanoparticle-Enhanced Raman Spectroscopy: Toward High Sensitivity and Broad Applicability. ACS Nano.

[41] Liu Y., Chui K.K., Fang Y., Wen S., Zhuo X., Wang J. (2024). Metal–organic framework-enabled trapping of volatile organic compounds into plasmonic nanogaps for surface-enhanced raman scattering detection. ACS Nano.

[42] Wu J., Li J., Liu X., Gong L., Chen J., Tang Z., Lin W., Mu Y., Lin X., Hong W. (2021). Unclonable photonic crystal hydrogels with controllable encoding capacity for anticounterfeiting. ACS Appl. Mater. Interfaces.

[43] Jiang X., Lai Y., Yang M., Yang H., Jiang W., Zhan J. (2012). Silver nanoparticle aggregates on copper foil for reliable quantitative SERS analysis of polycyclic aromatic hydrocarbons with a portable Raman spectrometer. Analyst.

[44] Huang Z., Liao T., Liu X., Wu J., Tang Z., Pan G., Liu H., Lin X., Lin W., Li Y. (2022). Modified ethylene/α-octene co-polymer composites based on dual toughening of hydrogen bonding network and nanospheres. Polym. Adv. Technol..

[45] Truong V.X., Bachmann J., Unterreiner A.N., Blinco J.P., Barner-Kowollik C. (2022). Wavelength-orthogonal stiffening of hydrogel networks with visible light. Angew. Chem. Int. Ed..

[46] Stanton M.M., Ducker R.E., MacDonald J.C., Lambert C.R., McGimpsey W.G. (2012). Super-hydrophobic, highly adhesive, polydimethylsiloxane (PDMS) surfaces. J. Colloid Interface Sci..

[47] Ismail A.E., Grest G.S., Heine D.R., Stevens M.J., Tsige M. (2009). Interfacial structure and dynamics of siloxane systems: PDMS− vapor and PDMS− water. Macromolecules.

[48] Chen Y., Cao J., Wei H., Wu Z., Wang X., Pei Y. (2021). Synthesis of polyvinyl alcohol/Ag electrospun nanofibers as highly efficient flexible SERS substrates. Vib. Spectrosc..

[49] Wang X.-Z., Hu Q., Gao M., Liu S., Luo J.-L. (2022). Tuning the subsurface oxygen of Ag2O-derived Ag nanoparticles to achieve efficient CO2 electroreduction to CO. Electrochim. Acta.

[50] Cao T., Zhou Y., Zhang J., Zhao B. (2025). Clean preparation of Ag@ Au nanoparticle-modified cotton fabric with durable antimicrobial properties by adsorption. Cellulose.

[51] Khan J., Naseem I., Bibi S., Ahmad S., Altaf F., Hafeez M., Almoneef M.M., Ahmad K. (2022). Green synthesis of silver nanoparticles (Ag-NPs) using Debregeasia salicifolia for biological applications. Materials.

[52] Liu G., Li K., Zhang Y., Du J., Ghafoor S., Lu Y. (2020). A facile periodic porous Au nanoparticle array with high-density and built-in hotspots for SERS analysis. Appl. Surf. Sci..

[53] Yu H., Chen Y., Wen Z., Wang R., Jia S., Zhu W., Song Y., Sun H., Liu B. (2024). Selective SERS Sensing of R6G Molecules Using MoS2 Nanoflowers under Pressure. Langmuir.

[54] Tang Z., Wu J., Liu X., Su Q., Yin X., Huang Z., Lin X., Lin W., Yi G. (2021). Au-Nanoparticle-Array/Aligned-Ag-Nanowire-Based Flexible Dual Plasmonic Substrate for Sensitive Surface-Enhanced Raman Scattering Detection. Part. Part. Syst. Charact..

[55] Yang B., Chen G., Ghafoor A., Zhang Y.F., Zhang X.B., Li H., Dong X.R., Wang R.P., Zhang Y., Zhang Y. (2023). Chemical Enhancement and Quenching in Single-Molecule Tip-Enhanced Raman Spectroscopy. Angew. Chem. Int. Ed..

[56] Yang T.-H., Ahn J., Shi S., Qin D. (2021). Understanding the role of poly (vinylpyrrolidone) in stabilizing and capping colloidal silver nanocrystals. ACS Nano.

[57] Badawy A.M.E., Luxton T.P., Silva R.G., Scheckel K.G., Suidan M.T., Tolaymat T.M. (2010). Impact of environmental conditions (pH, ionic strength, and electrolyte type) on the surface charge and aggregation of silver nanoparticles suspensions. Environ. Sci. Technol..

[58] Dimitriev O.P., Blank D.A., Ganser C., Teichert C. (2018). Effect of the polymer chain arrangement on exciton and polaron dynamics in P3HT and P3HT: PCBM films. J. Phys. Chem. C.

[59] Rekha C., Sameera S., Nayar V., Gopchandran K. (2019). Simultaneous detection of different probe molecules using silver nanowires as SERS substrates. Spectrochim. Acta Part A Mol. Biomol. Spectrosc..

[60] Xu T., Wang X., Huang Y., Lai K., Fan Y. (2019). Rapid detection of trace methylene blue and malachite green in four fish tissues by ultra-sensitive surface-enhanced Raman spectroscopy coated with gold nanorods. Food Control.

[61] Fateixa S., Nogueira H.I., Trindade T. (2018). Surface-enhanced Raman scattering spectral imaging for the attomolar range detection of crystal violet in contaminated water. ACS Omega.

