# Supplementary material for: A Composite Substrate of Ag Nanoparticle-Decorated Inverse Opal Polydimethylsiloxane for Surface Raman Fluorescence Dual Enhancement
Source: Polymers (Basel). 2025 Jul 21;17(14):1995. doi: 10.3390/polym17141995 (PMC12300014; doi:10.3390/polym17141995)
Supplement: Supplementary file 1 [file polymers-17-01995-s001.zip › polymers-3730467-supplementary.pdf]

polymers

MDPI

Article

Supplementary Materials: A Composite Substrate of Ag Nanoparticle-Decorated Inverse Opal Polydimethylsiloxane for Surface Raman Fluorescence Dual Enhancement

Zilun Tang [1,2], Hongping Liang [2,*], Zhangyang Chen [1], Jianpeng Li [1], Jianyu Wu [3], Xianfeng Li [1] and Dingshu Xiao [1,2,*]

[1] School of Chemistry and Materials Engineering, Huizhou University, Huizhou 516007, China; tangzl@hzu.edu.cn (Z.T.); 18928934633@163.com (Z.C.); lijp@hzu.edu.cn (J.L.); lxfeng@hzu.edu.cn (X.L.)
[2] Guangdong Provincial Key Laboratory of Electronic Functional Materials and Devices, Huizhou University, Huizhou 516007, China
[3] Science and Technology on Reliability Physics and Application of Electronic Component Laboratory, China Electronic Product Reliability and Environmental Testing Research Institute, Guangzhou 511370, China; wujianyu@ceprei.com
* Correspondence: lianghp@hzu.edu.cn (H.L.); hgyjy@hzu.edu.cn (D.X.)

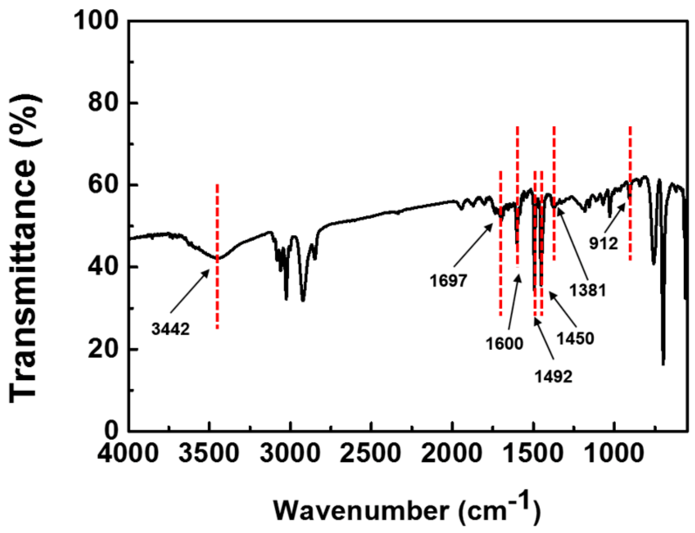

Figure S1. FTIR spectrum of PS nanoparticles.

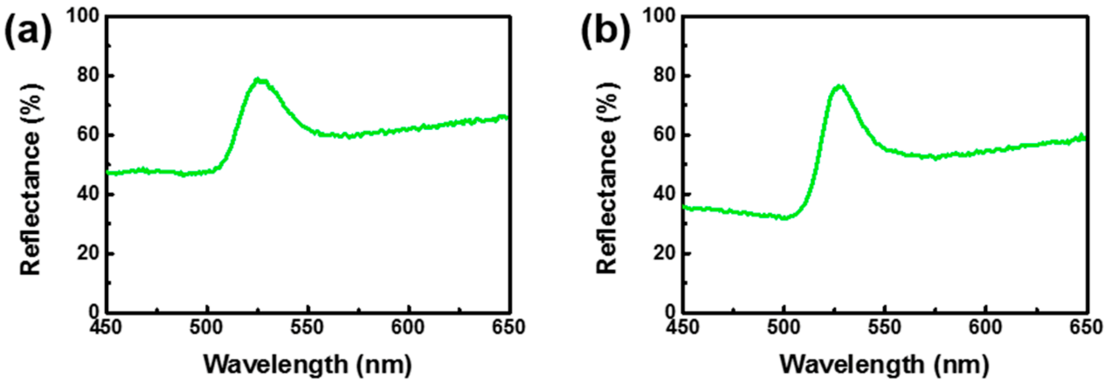

Figure S2. Reflection spectra of (a) PS nanoparticles after self-assembly and (b) PS/PDMS composite film.

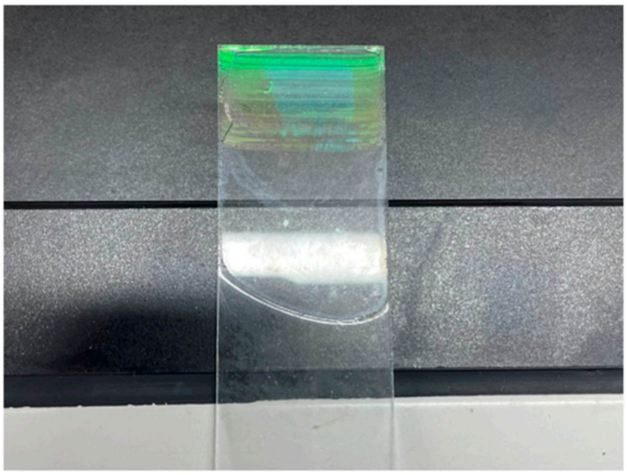

Figure S3. Photograph of the glass wafer after the transfer of PS photonic crystals.

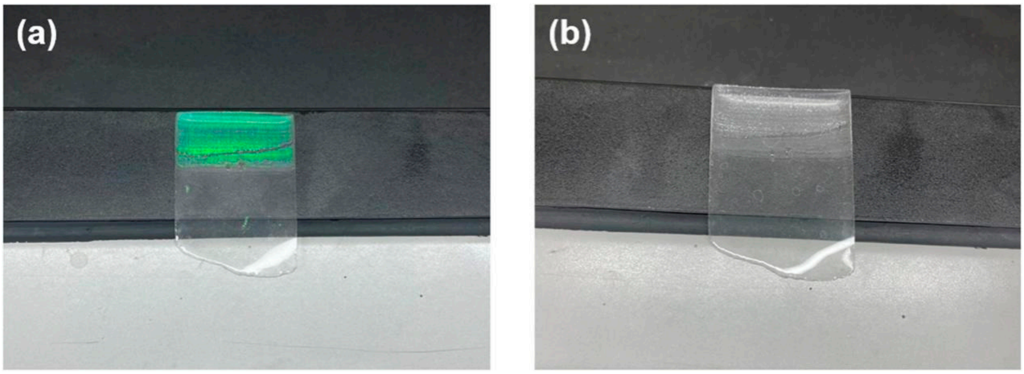

Figure S4. Photograph of PS / PDMS composite film (a) before and (b) after the etching of DMF.

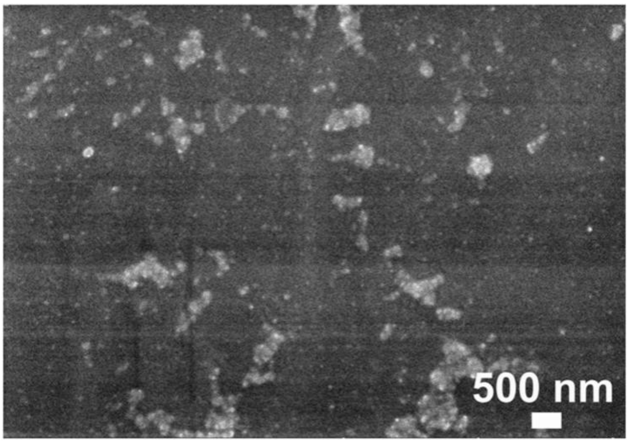

Figure S5. SEM image of Ag NP-decorated PDMS.

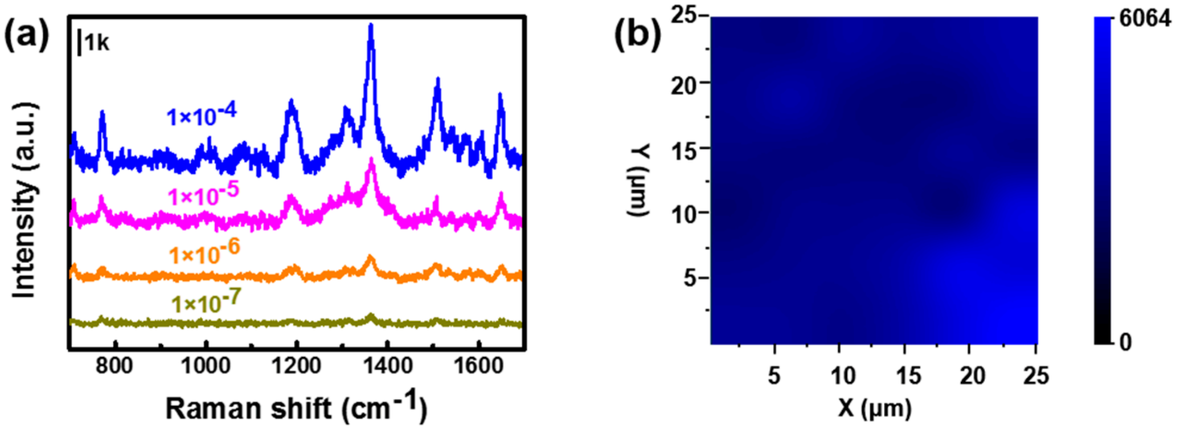

Figure S6. (a) Raman spectra of R6G with a concentration from 1×10^{-4} M to 1×10^{-7} M on AIOP composite substrate. (b) Raman mapping at the resolution of 5×5 pixels of R6G (1×10^{-4} M) on the AIOP composite substrate.

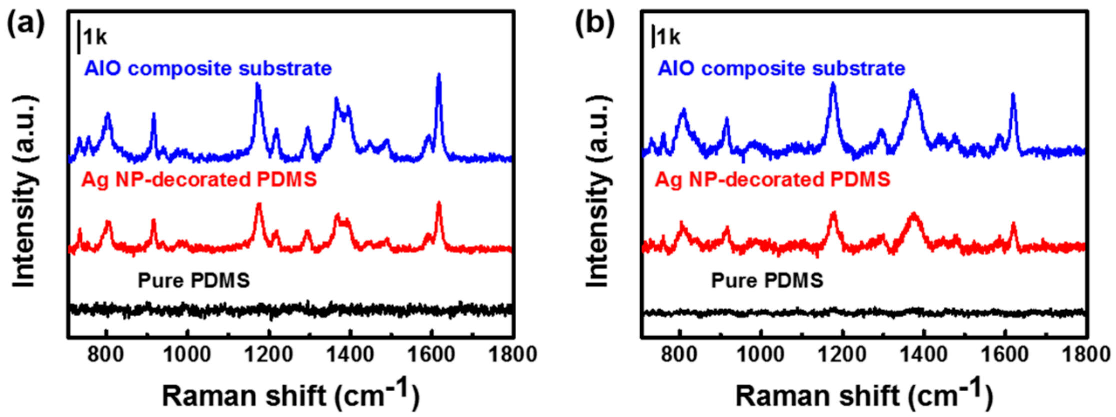

Figure S7. Raman spectra of (a) MG (1×10^{-4} M) and (b) CV (1×10^{-4} M) on pure PDMS, Ag NP-decorated PDMS, and AIOP composite substrate.

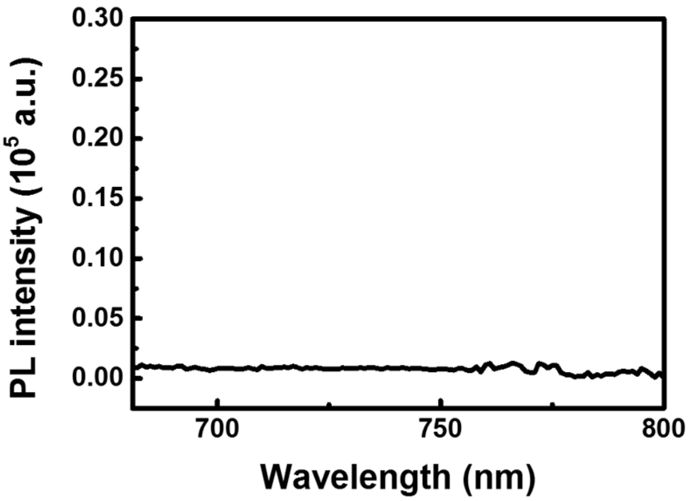

Figure S8. Fluorescence spectra of AIOP composite substrate ranging from 680 to 800 nm.

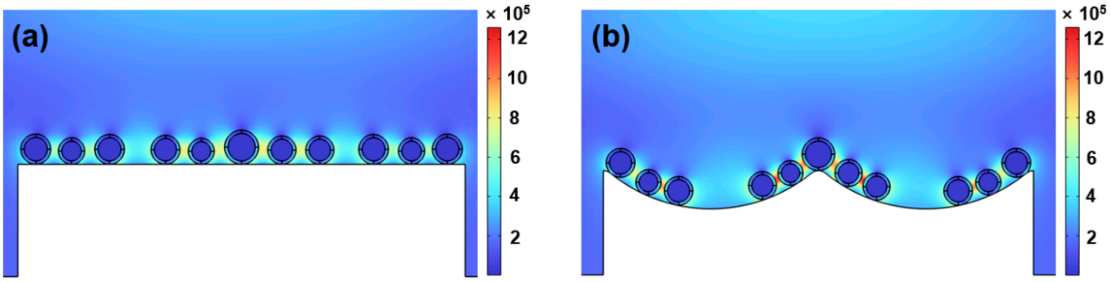

Figure S9. EM field distributions of cross-section of (a) Ag NP-decorated PDMS and (b) AIOP composite substrate under the excitation of 448 nm.

Table S1. Vibrational Models of the Raman peaks for R6G molecules [52, 53].

Raman Shift (cm^{-1})	Assignments
770	C−H out-plane bending
1187	C−H/N−H xanthene ring bending
1306	C=C stretching
1360	C−C xanthene ring stretching
1502	C−C xanthene ring stretching
1649	C−C in-plane stretching

Table S2. Vibrational Models of the Raman peaks for MG molecules [59, 60].

Raman Shift (cm^{-1})	Assignments
916	C−H out-of-plane bending
1171	C−H in-plane bending
1294	ring C−C stretching
1365	N-phenyl stretching
1589	ring C−C stretching
1614	ring C−C stretching

Table S3. Vibrational Models of the Raman peaks for CV molecules [61].

Raman Shift (cm^{-1})	Assignments
915	ring skeletal vibration of radical orientation
1171	ring C−H bending
1371	N-phenyl stretching
1531	ring C−C stretching
1629	ring C−C stretching

Disclaimer/Publisher's Note: The statements, opinions and data contained in all publications are solely those of the individual author(s) and contributor(s) and not of MDPI and/or the editor(s). MDPI and/or the editor(s) disclaim responsibility for any injury to people or property resulting from any ideas, methods, instructions or products referred to in the content.
